# Change for Life/*Cambia tu vida*: A Health Promotion Program Based on the Stages of Change Model for African Descendent and Latino Adults in New Hampshire

**Published:** 2006-06-15

**Authors:** Chris Smith, Andrew Ryan

**Affiliations:** New Hampshire Minority Health Coalition; Heller School for Social Policy and Management, Brandeis University, Waltham, Mass; At the time this work was done, Mr Ryan was with the Research and Evaluation Group, New Hampshire Minority Health Coalition, Manchester, NH

## Abstract

Studies have shown that diabetes and cardiovascular disease can be controlled and prevented through the modification of behavioral risk factors. The transtheoretical model of behavior change, also known as the s*tages of change* model, offers promise for designing behavior change interventions. However, this model has rarely been applied in group settings with minority communities. To address racial and ethnic disparities related to the risk for diabetes and cardiovascular disease, the New Hampshire REACH 2010 Initiative has designed and implemented Change for Life/*Cambia tu vida*, a health promotion program based on the stages of change model for African descendent and Latino residents of southern New Hampshire. The program guides participants through the five stages of change and provides resources to support healthy behavior change. We also sponsor periodic class reunions that help program graduates to maintain these healthy habits. This article describes curriculum development, participant feedback, and early pretest and posttest evaluation results from a standardized assessment.

## Reducing Risk Factors for Diabetes and Cardiovascular Disease Among Minority Populations

Diabetes and cardiovascular disease and the complications resulting from these diseases disproportionately affect racial and ethnic minority populations, particularly blacks and Latinos. The Racial and Ethnic Approaches to Community Health (REACH) 2010 Risk Factor Survey, conducted in 21 minority communities in the United States, showed that median prevalence rates of diabetes were 12.5% among blacks and 11.4% among Hispanics, compared with 6.1% among the general population in the United States (1). Similarly, median prevalence rates of cardiovascular disease were 9.4% among blacks and 8.3% among Latinos, compared with 7.6% among the general population (1). 

Although diabetes and cardiovascular disease pose serious health risks, several studies have shown that these diseases can be controlled and prevented through modification of risk factors (2-4). The Diabetes Prevention Program established that modification of eating and exercise habits decreases the probability that individuals with impaired glucose tolerance will develop type 2 diabetes (2). Similarly, evidence from the Framingham Heart Study indicates that overweight, smoking, lack of exercise, and unhealthy eating habits are all related to the development of heart disease (3) and that the modification of these risk factors can reduce mortality rates from heart disease (4).  

As the relationship between modifiable risk factors and the onset and progression of these diseases has become increasingly clear, the transtheoretical model of behavior change, more commonly known as the *stages of change* model (5), has been seen as a promising theoretical framework for behavior change interventions. The model posits that successful behavior change results when an individual progresses through five stages of change that culminate in the ability to maintain the desired behavior for the long term. Each of the following five stages in the model is defined in relation to when an individual plans to take action to change a behavior: 1) precontemplation — not intending to take action in the next 6 months; 2) contemplation — intending to take action in the next 6 months; 3) preparation — intending to take action in the next 30 days; 4) action —  has made overt changes for less than 6 months; and 5) maintenance — has made overt changes for more than 6 months (6). Individuals may move sequentially through the stages or, as is more common, may revert to an earlier stage one or more times during the process. The model also includes 10 *processes of change *that people use to move through the stages. There are five *experiential* processes (e.g., consciousness raising, self-reevaluation) that are used primarily in earlier stages and five *behavioral* processes (e.g., helping relationships, stimulus control) that are used primarily in later stages (5,6).

The stages of change model has broad applications for improving behavioral risk factors for both diabetes and hypertension. It can help participants both to acquire new healthy behaviors, such as exercise (7), stress management (8), and better diet (9), and to stop unhealthy habits such as smoking (10) and substance abuse (11). However, most interventions based on the model have been conducted in a clinical setting or, occasionally, through a home-based approach in which an intervention is tailored to an individual based on responses to an assessment tool (12). Very few interventions have been implemented in group settings in the community. Although the model has been used with African Americans and Latinos (13-15), the authors found only three studies of a group-based behavioral intervention using the stages of change model with minority participants (16-18). One evaluated a community-based program that was individually tailored to the stages of change of African American women and found that they were more likely to have reduced their fat intake three months later than were those in a randomly assigned control group (16).

The New Hampshire (NH) Minority Health Coalition, through its REACH 2010 Initiative, has designed a program that implements the stages of change model as a group-based intervention. Change for Life, or *Cambia tu vida*, is designed to help participants from minority communities modify behaviors that may put them at risk for diabetes and cardiovascular disease. Sponsored by the Centers for Disease Control and Prevention and first implemented in 2002, it is one of several programs offered by the NH REACH 2010 Initiative to address racial and ethnic health disparities in New Hampshire. (More information about the NH REACH 2010 Initiative is available from www.nhhealthequity.org/pro_reach.html.)

## Context

The Change for Life program serves the growing black and Latino population of Hillsborough County in southern New Hampshire. Hillsborough County includes Manchester and Nashua, the two largest cities in the state. The county's population is culturally diverse. An increasing number of residents are recent immigrants from Africa and the Caribbean, including Sudan, Nigeria, and Haiti. The REACH 2010 Initiative uses the term *African descendent* to acknowledge and welcome these participants in addition to African Americans. The Latino population is also diverse, with individuals from other U.S. states, Puerto Rico, the Dominican Republic, Mexico, Colombia (19), and other Latin American countries.

The REACH 2010 Risk Factor Survey (20) in 2004 documented economic and health disparities among blacks and Latinos in Hillsborough County as compared with New Hampshire overall. Although less than 20% of non-Hispanic white New Hampshire residents earned less than $25,000 per year according to the NH Behavioral Risk Factor Surveillance System (BRFSS) (21), 44% of blacks and 54% of Latinos in Hillsborough County were in that income category in the REACH 2010 survey. Similarly, only 7% of non-Hispanic white New Hampshire residents had not finished their high school education, compared with 14% of blacks and 43% of Latinos in Hillsborough County. Fewer blacks (34%) or Latinos (37%) were in the normal weight range (based on body mass index) than the 43% of New Hampshire respondents to the BRFSS. Minorities were also less physically active, with 18% of blacks and 27% of Latinos reporting no moderate or vigorous activity in a usual week compared with 10% of all New Hampshire residents (20,21). Although prevalence rates of hypertension and diabetes are comparable among populations, blacks and Latinos who have been diagnosed with either condition are less likely to receive treatment. Among adults with diagnosed hypertension, 55% of blacks and Latinos in Hillsborough County take antihypertensive medication, compared with 77% of New Hampshire overall (21). Similarly, 55% of black and Latino respondents with diagnosed diabetes in the REACH 2010 survey reported having had a hemoglobin A1c test in the past year, compared with nearly 90% of New Hampshire BRFSS respondents.

The physical environment may contribute to health disparities. The neighborhoods in Manchester and Nashua with predominantly black or Latino residents lack access to healthy foods. Although there are two large supermarkets in Manchester and one in Nashua, they are not easily accessible to many low-income residents who do not drive or own vehicles. Instead, neighborhood markets or "superettes" are more common, and they tend to have more expensive food and a limited selection of fresh fruits and vegetables. Several studies have linked access to food stores to fruit and vegetable consumption (22,23) and have shown that lack of access can disproportionately affect minority communities (24). In addition, the long, dark New Hampshire winters and the lack of affordable recreational facilities for low-income residents can discourage physical activity, as has been shown among older adults in minority populations (25).

These economic and health disparities are occurring in minority populations that are much younger than the rest of New Hampshire. According to the 2000 U.S. census, the median age of New Hampshire's non-Hispanic whites is 37.6 years, compared with 28.3 years among blacks and 24.2 years among Latinos. More than 60% of black and Latino respondents in the REACH 2010 survey were younger than 40 years, compared with 30% of New Hampshire BRFSS respondents (20,21). Disparities in rates of chronic disease and the risk factors that lead to these diseases are likely to increase as New Hampshire's younger minority communities age.

## Program Development and Implementation

The Change for Life/*Cambia tu vida* program was developed to reduce health disparities by addressing the increased risk of developing diabetes and hypertension among African descendent and Latino residents of Hillsborough County. Table 1 provides the timeline for designing, pilot testing, implementing, and evaluating the program.

### Curriculum

The NH REACH 2010 Initiative team designed the curriculum for Change for Life based on the stages of change model described in the book by Prochaska et al, *Changing for Good* (5). The team also reviewed health promotion programs identified by Pro-Change Behavior Systems, Inc (West Kingston, RI) on its Web site (6). The stages of change model was adapted in several ways to be culturally appropriate for Latino and African descendant participants. For example, instead of emphasizing independent self-help, Change for Life/*Cambia tu vida* was designed to provide group support for individual change. The program also encourages participants to choose which health behaviors to change rather than counseling them about what they should change about their behavior. Finally, the program educates participants about the stages of change so that they are able to apply the model in the future to other aspects of their lives.

After the curriculum was drafted, it was reviewed by NH REACH 2010 cultural advisors from the targeted communities. They recommended adding graphics to the materials and revising some of the language, not only to reach low-literacy participants but also to include more culturally relevant examples in the curriculum. After these changes were made, the curriculum and class materials were translated into Spanish. Both the Spanish and English curricula were then reviewed by health literacy experts. The facilitator's manual was revised to a sixth-grade reading level, and the participant workbook was written at a fourth-grade reading level. The curriculum was then pilot tested in both communities and revised again based on the results.

### Intervention 

The Change for Life program consists of six 2-hour classes held weekly, followed by periodic group support meetings after the series of classes is completed. The first Change for Life class series began in November 2002, and the *Cambia tu vida* classes began in January 2003. *Cambia tu vida* is facilitated by a native speaker of Spanish. At the beginning of each class series, participants are required to choose at least one of the modifiable risk factors for diabetes and cardiovascular disease that they would like to change. Participants focusing on different modifiable risk factors and in different stages of change are often included in the same class to help participants learn from each other how to use the stages of change model.

**Figure 1:**
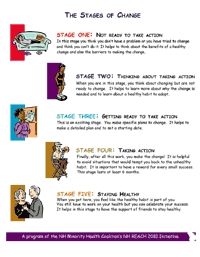
Handout summarizing the curriculum for participants in the Change for Life program, New Hampshire Racial and Ethnic Approaches to Community Health (REACH) 2010 Initiative.

**Figure 2:**
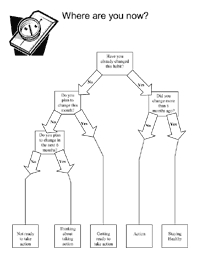
Stages of change decision tree used in the Change for Life program, New Hampshire Racial and Ethnic Approaches to Community Health (REACH) 2010 Initiative.

In each class, the facilitator teaches participants about one or two of the five stages of change. Figure 1 shows a handout summarizing the curriculum for participants. The curriculum includes group exercises tailored to the stage being discussed that encourage facilitators and participants to share their experiences with current and previous efforts at behavior change. Participants are taught how to identify their triggers for the unhealthy habit they want to change, recognize the barriers to change, and solicit the support they need to begin to make the change. Participants set realistic goals for their behavior change and reward themselves for their progress in taking overt actions in their change plan. Each week they are also encouraged to track their progress through the stages of change by using a decision tree (Figure 2). The decision tree is also used in the participant workbook and in evaluation assessments.

### Facilitator training

For the first year, the Change for Life and *Cambia tu vida* classes were facilitated by NH REACH 2010 staff. After that, the program shifted to a train-the-trainer model, and community members were trained to facilitate the classes. Facilitator training is conducted in a single 4-hour session that includes a review of the curriculum and instruction on group facilitation techniques. Community facilitators receive a facilitator's manual, designed and tested by NH REACH 2010 staff, with information on leading the groups as well as detailed lesson plans for each session.

Facilitators are paid to organize and teach Change for Life or *Cambia tu vida* classes in their communities. NH REACH 2010 provides facilitators with mentoring and support to recruit participants, conduct the classes, and collect data for the evaluation. Series taught by community facilitators are usually held at the homes of facilitators or participants. To date, four train-the-trainer sessions have been held, two in each community; 11 Latino and 16 African descendent community members have been trained to teach these classes. 

### Recruitment 

The first participants in the program were recruited from other NH REACH 2010 Initiative interventions. Later, as the train-the-trainer model was implemented, participants were also recruited by community facilitators, through staff community outreach, from referrals by partner organizations, and by word of mouth, frequently from program graduates. Classes are held in both Manchester and Nashua, with transportation and childcare provided.

From November 2002 through May 2005, 46 African descendents and 127 Latinos attended Change for Life or *Cambia tu vida* classes. Thirty-four of the African descendent participants and 91 of the Latino participants completed the program, for a completion rate of 72%. 

### Evaluation and data analysis

A quasi-experimental design for evaluation was implemented in October 2003. Participants were asked to report their stage of change at baseline, immediately after the last class, and 3 months after the last class using the decision tree (Figure 2). The nonparametric Wilcoxon matched-pairs signed rank test was used to test whether there was significant advancement through the stages of change by participants. The matched-pair comparisons were performed among all participants who had completed both of the assessments (i.e., baseline and last class or baseline and 3-month follow-up).

Qualitative information was obtained through semistructured interviews with NH REACH 2010 program coordinators and group discussions with participants at the close of the final session of the class. Staff were asked about how the intervention was developed and implemented, their perceptions of the impact of the class on participants' behavior change, and their perceptions of the factors that contributed to the strengths and weaknesses of the intervention. Participants were asked to provide general feedback about the program and how it might be improved in the future.

## Consequences

### Description of participants

From October 2003 through May 2005, 18 African descendent participants and 74 Latino participants responded to the baseline assessment. Of these 92 participants, 88 completed the class. Eleven African descendent participants and 40 Latino participants (total = 51) completed the 3-month follow-up assessment in time to be included for this article, for a preliminary follow-up rate among those who had graduated before March 2005 of 93% for African descendent graduates and 71% for Latino graduates.

Most of the participants who responded to the baseline assessment were women younger than 40 years (Table 2). Most were immigrants from Latin American countries such as Mexico and the Dominican Republic, although more than half of the participants had been born in the United States or had been in the United States for 5 or more years. Half of the participants had annual household incomes less than $25,000, and about 30% had not graduated from high school. Approximately 16% had been diagnosed with diabetes, and 24% had hypertension. Most chose diet (77%), exercise (52%), or both as the health habits they wanted to change by participating in the program. A few participants (14%) focused on reducing their cigarette smoking or alcohol consumption.

### Change among program participants

Table 3 shows matched-pair comparisons of participant stage of change status at baseline compared with the last class and at baseline compared with the 3-month follow-up. Forty-nine participants who were trying to improve their diets responded to the baseline and last class assessments. Of the participants who chose diet as their focus, 37% were in the first two stages (precontemplation or contemplation) at baseline, 39% were in the preparation stage, and 24% reported they were already taking action at baseline. These participants were significantly more likely to be in a more advanced stage of change after completing the class series than they had been before. For the 35 participants who also completed the 3-month follow-up assessment, responses on the stages of change measures reveal that participants continued their advances. The reported stages of change for the participants who wanted to exercise more were similarly distributed at baseline. Similar advances through the stages of change were reported by participants who focused on exercise or stress, although changes for these behaviors reported at 3-month follow-up are not statistically significant. When considered together, program participants showed significant progress to later stages of change at the last class and at 3-month follow-up.

### Participant feedback

Table 4 summarizes perceptions at the end of class and at the 3-month follow-up. Most participants who completed the assessment at the end of the last class reported that the program had been helpful. More than half of participants reported that they had made progress toward changing their health habits in the 3-month follow-up assessment. Discussions at the end of the class indicated that participants had benefited from the program. Participants reported becoming more aware of unhealthy behavior. For example, one participant explained: 

Since I've been coming to this group, . . . I can honestly say that I'm seeing things from a different perspective. Now when I'm doing something, no matter what it is, I get this awareness about this meeting. . . . [I now feel] I am accountable for the decisions and the actions that I make.

Participants also indicated the importance of support for behavior change. Another participant said:

I think that I have changed a lot because I used to eat a lot of things that I shouldn't eat. . . . Because of my [diabetes], I have to eat different foods and I wouldn't [before the class]. I would go and buy cake, and cake was bad for me, but I would eat it anyway, and I would eat the whole thing because since I don't have anyone to share it with or to give it to, I would eat it all. But I thank God, and thank you guys for all your help because you guys give these terrific classes that do so much good and help so many people, and I hope that this is not the last time that you guys give these classes because it does a lot of people very good.

Participant feedback also indicated a desire for continued support after the intervention. During the 3-month follow-up assessments, some participants reported having lost their momentum for behavior change once the class was over.

## Interpretation

Early evaluation results indicate that the Change for Life/*Cambia tu vida* intervention helped participants to make progress toward their goals of behavior change and created a better awareness of personal behavior in a fun, supportive, and culturally appropriate setting. In addition, the train-the-trainer program helped to empower participants to take an active role in improving the health of their communities. To respond to participant requests for continued support after the intervention, NH REACH 2010 staff sponsor class reunions that offer advice on nutrition and enlist the help of local fitness centers to provide reduced rates to program graduates.

The Change for Life/*Cambia tu vida* program has been more readily implemented among Latinos than among African descendents in New Hampshire. The home-based, group support model appears to be more appealing to Latino residents, especially women. A faith-based approach has been found to be more appealing to African descendents. One of the most successful facilitators of the Change for Life classes is an African American minister whose church serves a predominantly black neighborhood in a city center of Hillsborough County. 

When Change for Life was first implemented, the program evaluation was exclusively qualitative and aimed primarily at collecting information about participant satisfaction. Several months later, a quasi-experimental evaluation design was implemented to strengthen the outcome evaluation. The newer evaluation uses a pretest–posttest design with a comparison group of community members who are not randomly assigned but who have chosen not to participate in the program. Both participants in the intervention and members of the comparison group are administered assessments during the course of a year and are weighed and have their blood pressure taken each time. The assessments for both groups are conducted at baseline, 3 months later, and 1 year after baseline. The intervention and this evaluation are expected to continue through spring 2007. Our early findings indicate that African descendant and Latino participants are reporting progress toward their goals. Self-reported advancement through the stages of change suggest that participants have learned about the model and are applying it to their health behavior. Subsequent analysis will compare their self-reports with those of the comparison group to evaluate whether these behavioral intentions have resulted in the adoption of healthier habits in the months following participants' completion of the Change for Life/*Cambia tu vida *program.

## Figures and Tables

**Table 1:** Timeline of Change for Life/*Cambia tu vida *Program, New Hampshire Racial and Ethnic Approaches to Community Health (REACH) 2010 Initiative

**Date**	**Activity**
Spring 2002	Program curriculum developed based on stages of change model (5)
Summer 2002	Draft of curriculum reviewed by cultural advisors and piloted with community members
Fall 2002	Staff facilitators trained
November 2002	First Change for Life class conducted with African descendent participants
January 2003	First *Cambia tu vida* class conducted with Latino participants
June 2003	Train-the-trainers program begun to train community facilitators to teach classes
October 2003	Before/after evaluation design implemented with participants and community comparison group

**Table 2:** Description of Change For Life/*Cambia tu vida* Program Participants (N = 88) From Baseline Assessments, New Hampshire, October 2003–May 2005

**Characteristic**	**No. of Participants (%)**
**Sex**	
Male	22 (25)
Female	66 (75)
**Age, y**	
18-24	13 (14.8)
25-39	35 (39.8)
40-64	38 (43.2)
≥65	2 (2.3)
**Ethnic group**	
African American	9 (10.2)
African immigrant or refugee	9 (10.2)
Latino	70 (79.5)
**Years in United States**	
Born in U.S.	11 (12.5)
Less than 1	3 (3.4)
1 or more but less than 5	30 (34.1)
5-10	16 (18.2)
More than 10	28 (31.8)
**Annual income, $**	
<10,000	9 (10.2)
10,000-24,999	35 (39.8)
25,000-49,999	19 (21.6)
≥50,000	11 (12.5)
Don't know or refused	14 (15.9)
**Education**	
Less than 9th grade	16 (18.2)
Some high school (≥9th grade)	10 (11.4)
High school diploma or graduate equivalency degree	22 (25.0)
Some college	27 (30.7)
College graduate or more	13 (14.8)
**Chronic disease**	
Diabetes	14 (15.9)
Hypertension	21 (23.9)
**Area of focus at baseline^a^**	
Smoking	10 (11.4)
Alcohol consumption	2 (2.3)
Diet	68 (77.3)
Exercise	46 (52.3)
Stress	36 (40.9)

a Participants could choose more than one area for change.

**Table 3:** Pair-Comparison of the Distribution of Stages of Change Among Participants in Change for Life/*Cambia tu vida* Program at Baseline and Follow-up Assessments, New Hampshire, October 2003–May 2005

**Area of Focus and Stage of Change**	**Baseline and at Last Class (N = 67)^a^**			**Baseline and at 3-Month Follow-up (N = 46)^b^**		

	**Baseline No. (%)**	**Follow-up No. (%)**	***P* Value^c^**	**Baseline No. %**	**Follow-up No. (%)**	***P* Value^c^**
**Diet**						
Precontemplation	2 (4.1)	0	<.001	1 (2.9)	0	.002
Contemplation	16 (32.7)	3 (6.1)		13 (37.1)	9 (25.7)	
Preparation	19 (38.8)	15 (30.6)		13 (37.1)	2 (5.7)	
Action	9 (18.4)	29 (59.2)		7 (20.0)	20 (57.1)	
Maintenance	3 (6.1)	2 (4.1)		1 (2.9)	4 (11.4)	
**Exercise**						
Precontemplation	0	0	.04	1 (4.5)	1 (4.5)	.06
Contemplation	6 (27.3)	2 (9.1)		9 (40.9)	6 (27.3)	
Preparation	10 (45.5)	9 (40.9)		5 (22.7)	4 (18.2)	
Action	4 (18.2)	10 (45.5)		5 (22.7)	7 (31.8)	
Maintenance	2 (9.1)	1 (4.5)		2 (9.1)	4 (18.2)	
**Stress**						
Precontemplation	0	0	<.001	0	0	.06
Contemplation	7 (33.3)	3 (14.3)		4 (33.3)	4 (33.3)	
Preparation	12 (57.1)	4 (19.0)		6 (50.0)	0	
Action	2 (9.5)	13 (61.9)		2 (16.7)	6 (50.0)	
Maintenance	0	1 (4.8)		0	2 (16.7)	
**All areas**						
Precontemplation	2 (3.0)	0	<.001	1 (2.2)	0	<.001
Contemplation	22 (32.8)	6 (8.6)		16 (34.8)	11 (23.9)	
Preparation	26 (38.8)	20 (28.6)		18 (39.1)	3 (6.5)	
Action	13 (19.4)	40 (57.1)		9 (19.6)	21 (45.7)	
Maintenance	4 (6.0)	4 (5.7)		2 (4.3)	11 (23.9)	

a For diet, n = 49; for exercise, n = 22; for stress, n = 21. Participants could choose more than one area for change.

b For diet, n = 35; for exercise, n = 22; for stress, n = 12. Participants could choose more than one area for change.

c Test of statistical significance was one-tailed Wilcoxon matched-pair signed rank test.

**Table 4:** Change for Life/*Cambia tu vida* Participants' Perceptions of Intervention at the End of Class and at 3-Month Follow-up, New Hampshire, October 2003–May 2005

**Indicator**	**No. (%)**
**End of class**	
Helpfulness of the class (n = 72)	
Not helpful or somewhat helpful	6 (8.3)
Quite helpful	15 (20.8)
Very helpful	51 (70.8)
Helpfulness of the intervention workbook (n = 70)	
Not helpful or somewhat helpful	3 (4.3)
Quite helpful	23 (32.9)
Very helpful	44 (62.9)
**3-Month follow-up**	
Progress made toward changing behavior (n = 48)	
No progress or some progress	19 (39.6)
Quite a bit of progress	20 (41.7)
A lot of progress	9 (18.8)
Frequency of reading workbook (n = 49)	
Never	6 (12.2)
Rarely	10 (20.4)
Sometimes	30 (61.2)
Often	3 (6.1)
