# Supplementary material for: Change for Life/Cambia tu vida: A Health Promotion Program Based on the Stages of Change Model for African Descendent and Latino Adults in New Hampshire
Source: Prev Chronic Dis. 2006 Jun 15;3(3):A105. (PMC1637793)
Supplement: Supplementary file 1 [file 05_0218_01.pdf]

Figure 1. Change for Life Curriculum Summary.

THE STAGES OF CHANGE

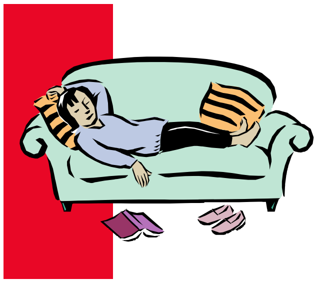

STAGE ONE: NOT READY TO TAKE ACTION

In this stage you think you don't have a problem or you have tried to change and think you can't do it. It helps to think about the benefits of a healthy change and also the barriers to making the change.

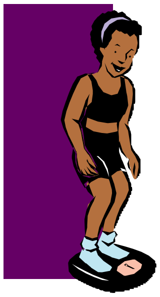

STAGE TWO: THINKING ABOUT TAKING ACTION

When you are in this stage, you think about changing but are not ready to change. It helps to learn more about why the change is needed and to learn about a healthy habit to adopt.

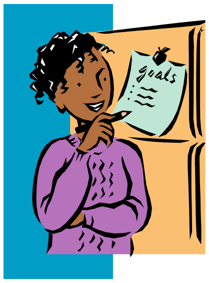

STAGE THREE: GETTING READY TO TAKE ACTION

This is an exciting stage. You make specific plans to change. It helps to make a detailed plan and to set a starting date.

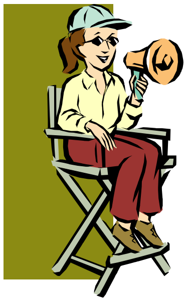

STAGE FOUR: TAKING ACTION

Finally, after all this work, you make the change! It is helpful to avoid situations that would tempt you back to the unhealthy habit. It is important to have a reward for every small success. This stage lasts at least 6 months.

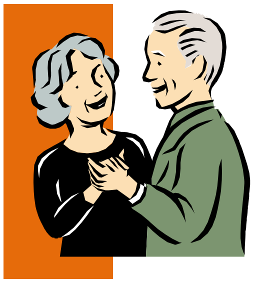

STAGE FIVE: STAYING HEALTHY

When you get here, you feel like the healthy habit is part of you. You still have to work on your health but you can celebrate your success. It helps in this stage to have the support of friends to stay healthy.
