# Supplementary figures and images for: Change for Life/Cambia tu vida: A Health Promotion Program Based on the Stages of Change Model for African Descendent and Latino Adults in New Hampshire
Source: Prev Chronic Dis. 2006 Jun 15;3(3):A105. (PMC1637793)

Figure 2. Stages of Change Decision Tree from the Change for Life Program.

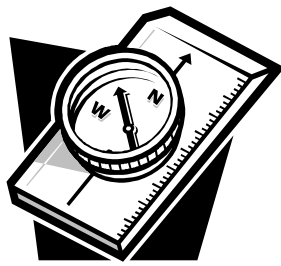

Where are you now?

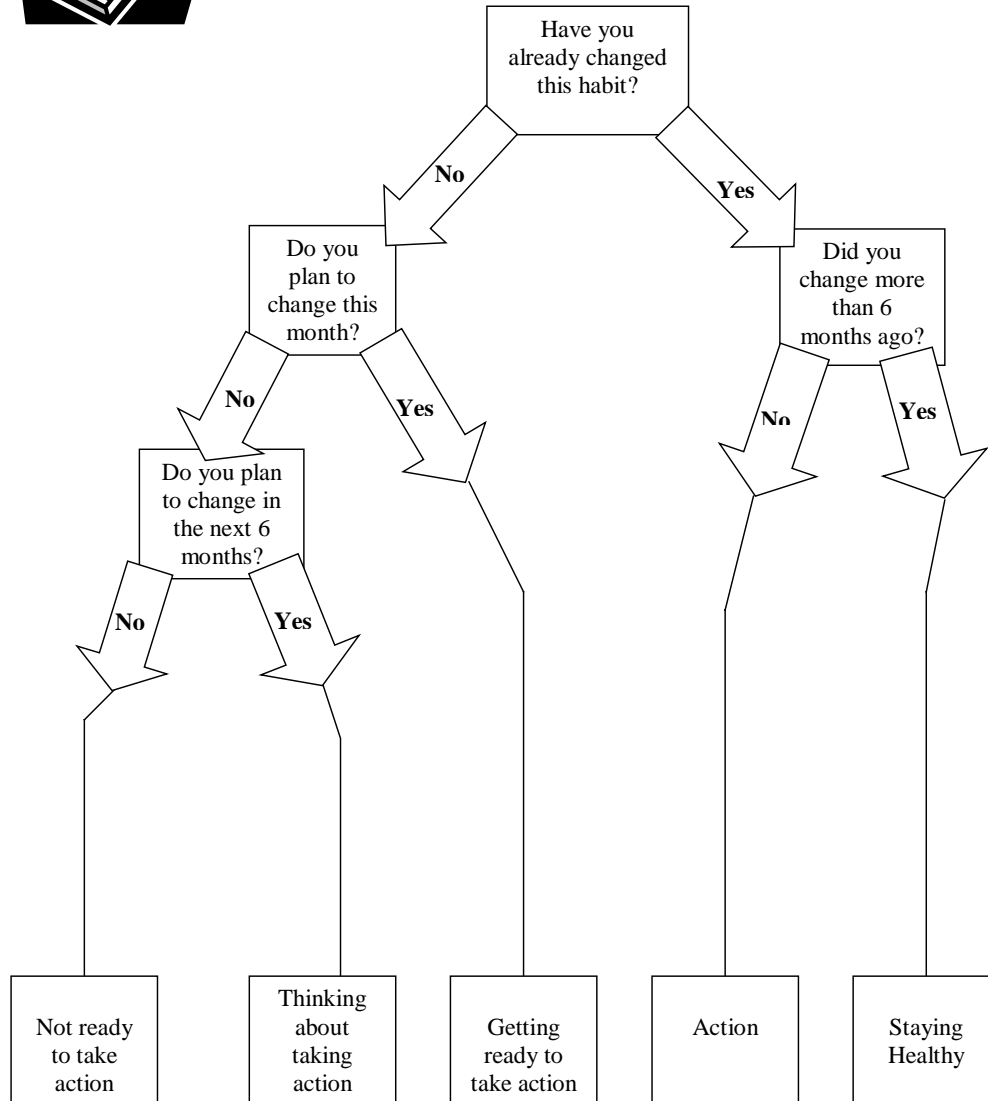

Supplement: Supplementary file 2 [file 05_0218_02.pdf]
